# Quasi-static image-based immersed boundary-finite element model of left ventricle under diastolic loading

**DOI:** 10.1002/cnm.2652

**Published:** 2014-05-28

**Authors:** Hao Gao, Huiming Wang, Colin Berry, Xiaoyu Luo, Boyce E Griffith

**Affiliations:** 1 School of Mathematics and Statistics, University of GlasgowGlasgow, UK; 2 School of Civil Engineering, Xinjiang UniversityXinjiang, China; 3 Institute of Cardiovascular and Medical Sciences, University of GlasgowGlasgow, UK; 4 Leon H. Charney Division of Cardiology, Department of Medicine, New York University School of MedicineNew York, New York, USA; 5 Department of Mathematics, Courant Institute of Mathematical Sciences, New York UniversityNew York, New York, USA

**Keywords:** immersed boundary method, finite element method, ventricular mechanics, hyperelasticity, structure-based constitutive model, left ventricle

## Abstract

Finite stress and strain analyses of the heart provide insight into the biomechanics of myocardial function and dysfunction. Herein, we describe progress toward dynamic patient-specific models of the left ventricle using an immersed boundary (IB) method with a finite element (FE) structural mechanics model. We use a structure-based hyperelastic strain-energy function to describe the passive mechanics of the ventricular myocardium, a realistic anatomical geometry reconstructed from clinical magnetic resonance images of a healthy human heart, and a rule-based fiber architecture. Numerical predictions of this IB/FE model are compared with results obtained by a commercial FE solver. We demonstrate that the IB/FE model yields results that are in good agreement with those of the conventional FE model under diastolic loading conditions, and the predictions of the LV model using either numerical method are shown to be consistent with previous computational and experimental data. These results are among the first to analyze the stress and strain predictions of IB models of ventricular mechanics, and they serve both to verify the IB/FE simulation framework and to validate the IB/FE model. Moreover, this work represents an important step toward using such models for fully dynamic fluid–structure interaction simulations of the heart. © 2014 The Authors. *International Journal for Numerical Methods in Engineering* published by John Wiley & Sons, Ltd.

## 1. INTRODUCTION

Cardiac diseases have been a major public health burden in industrialized countries for more than a century and are now the leading causes of morbidity and mortality worldwide. Despite intense effort, however, the biomechanics of heart disease and heart failure remain incompletely understood. It is well known that alterations in myocardial stress and strain distributions can have a significant impact on maladaptive processes such as hypertrophy 1,2, but it is not possible to measure directly intramural stress distributions in patients. Three-dimensional stress and strain distributions are readily provided by computational models of the heart, however, and such models therefore could assist in patient risk stratification and inform clinical decision making 3. An improved understanding of ventricular biomechanics is also important for optimizing medical therapies and surgical procedures aimed at restoring normal heart function 4.

The development of realistic computational models of the heart is complicated by several factors. The heart is a multiphysics system in which the contractions of the myocardium are stimulated and coordinated by the electrophysiology of the heart. The passive and active mechanical properties of the myocardium are anisotropic and nonlinear, and the heart experiences large deformations resulting both from diastolic filling and also from systolic contraction. In addition, the heart is fundamentally a fluid–structure system, and a fully dynamic computational model of the heart must account for the substantial inertia of both the muscular heart wall and the blood. Finally, there are significant intersubject differences in cardiac anatomy and physiology that must be incorporated into fully subject-specific computational models. For a recent survey of approaches to patient-specific modeling of cardiac biomechanics, see ref. 5 .

Because of the complex anatomical geometry, large deformations, and nonlinear material response of the heart, most detailed models of cardiac mechanics have used nonlinear finite element (FE) methods. Nash and Hunter 6 developed an FE framework for large deformation heart simulation using the pole zero constitutive law. A subsequent study by Stevens *et al*. 7 suggested that the pole-zero law might need to be reformulated to achieve a better separation of material parameters associated with deviatoric stresses. Vetter and McCulloch 8 modeled the rabbit left ventricle (LV) in diastole using a three-dimensional FE method, and a good agreement with experimental measurement was found. Using a three-dimensional finite deformation model, Usyk *et al*. 9 studied the effects of the laminar fiber architecture on regional stress and strain.

Since the 1990's, various studies have demonstrated that the layered organization of the ventricular myofibrils results in an orthotropic passive material response 10,11. Relatively few studies have used orthotropic material laws for LV simulations, however. Instead, the most earlier work has described the myocardium as transversely isotropic 12,13. The Holzapfel–Ogden model of the ventricular myocardium 10 provides a description of the orthotropic and hyperelastic behavior of cardiac muscle that is based on strain invariants that reflect the structure of the myocardium. Among the first organ-scale simulations to use this model were by Göktepe *et al*. 14, who used it with an FE model of the heart with an idealized biventricular geometry.

In earlier work, we also used the Holzapfel–Ogden strain-energy functional to model ventricular mechanics. We developed a realistic human left ventricular geometry derived from clinical magnetic resonance (MR) imaging data along with a rule-based fiber architecture. Using this model along with a nonlinear FE method for the structural mechanics, we studied the effects of changes in fiber organization and material properties on diastolic mechanics, and we demonstrated that changes to the fiber orientations have substantially larger effects on the passive response of the myocardium than changes to the sheet orientations 15. We also showed that the effects of the residual stress, determined either from measured residual strains or by the opening-angle method, can be easily incorporated in a modified Holzapfel–Ogden model 16.

Dynamic fluid–structure interaction (FSI) models of the heart have also been developed. Perhaps the most widely used approach to construct such models is to use body-conforming discretizations of the fluid and structure, for example, via an arbitrary Lagrangian–Eulerian (ALE) formulation 17–20. Watanabe *et al.* 17 developed an FSI model of the LV using an ALE FE method with strong coupling that also included a description of the electrophysiology of the LV. More recently, Nordsletten *et al.* 18 developed an FE framework for simulating blood flow and myocardial dynamics in both the diastolic and systolic phases of the cardiac cycle via a nonconforming ALE FE scheme. Their method has been used to study the effects of left ventricular assist devices on the heart 19 and to investigate the diastolic function of patients with hypoplastic left heart syndrome 20.

Fluid–structure interaction methods involving body-fitted grids generally require dynamic mesh generation for problems that involve large structural deformations and this requirement substantially complicates the implementation of such schemes. The immersed boundary (IB) method 21 offers an alternative to body-fitted methods that avoid the need for dynamic mesh generation. The IB method is a general approach to modeling systems in which an incompressible elastic structure is immersed in a viscous incompressible fluid. Within the IB framework, the momentum, viscosity, and incompressibility of the coupled fluid–structure system are described in Eulerian form, and the structural deformations, stresses, and forces are described in Lagrangian form. Integral equations with Dirac delta function kernels couple the Eulerian and Lagrangian variables, and when the continuous equations are discretized, the singular delta function is replaced by a carefully constructed regularized version of the delta function. A key advantage of the IB approach to FSI is that it does not require the construction of conforming discretizations of the fluid and solid. This approach is therefore extremely well-suited for applications such as cardiac dynamics that involve large structural deformations.

Conventionally, many of the structural models used with the IB method have described the elasticity of the immersed body using systems of elastic fibers. Such fiber models have been used to develop a variety of biofluid dynamics models, including detailed three-dimensional models of the heart 22–28 and its valves 29–32. Although fiber-based elasticity models are well-suited to describing anisotropic tissues such as the ventricular myocardium, it is challenging to use fiber models along with constitutive models, such as the Holzapfel–Ogden model 10, that account for the experimentally observed orthotropic properties of the ventricular myocardium. Further, in ventricular mechanics, stress and strain are important biomarkers 2, and despite extensive work on simulating cardiac dynamics by the IB method, we are aware of no previous studies that have assessed the accuracy of the stress and strain predictions of IB models of cardiac mechanics.

The IB method is not restricted to fiber-based elasticity models, and various extensions of the IB method have been introduced that use FE-based structural models 33–39. In this study, we apply one of these schemes, a hybrid finite difference-finite element IB method 38 to simulate left ventricular biomechanics under diastolic loading conditions, and we present an initial verification of the accuracy of the predicted stress and strain distributions by comparing the model results to those generated by a well-established FE solver. As in our earlier work using a purely structural nonlinear FE method 15, here, we use the structure-based hyperelastic ventricular model of Holzapfel and Ogden 10 along with an anatomical geometry derived from clinical MR imaging data obtained from a healthy human subject and a rule-based myocardial fiber structure. Numerical predictions of the IB/FE LV model are shown to be in good qualitative and quantitative agreement with results from the earlier FE-based LV model, and the predictions of both models are shown to be in good agreement with previous computational and experimental data. We also demonstrate that improved numerical predictions of the intramural strain and stress can be obtained by employing straightforward modifications of the constitutive model that effectively redistribute part of the hydrostatic pressure from the Eulerian grid to the Lagrangian mesh.

Although the present study considers only the quasi-static mechanics of the LV under diastolic loading conditions, it is nonetheless among the first to employ the IB method to simulate cardiac mechanics along with realistic descriptions of cardiac anatomy and physiology. Moreover, our results serve to verify the IB/FE methodology and also provide an initial validation of this IB/FE model of ventricular mechanics. Our results also are among the first to demonstrate that the IB method is capable of producing quantitatively accurate stress and strain distributions for realistic models of the heart and is thereby a suitable framework for developing realistic FSI models of the heart.

## 2. METHODOLOGY

In this section, we give the details of developing an IB/FE LV model based on a subject-specific LV geometry from clinical imaging. We begin with a brief introduction of the IB formulation and the constitutive model of the passive myocardium. We then discuss modifications of the structural stress tensor that improve the accuracy of the computed structural stresses, as demonstrated empirically in Section 3. Next, we give an overview of the numerical methods, including the spatial discretization and time-stepping schemes. We subsequently describe the reconstruction of a human left ventricular model from MRI data. We conclude by describing the benchmark nonlinear FE LV model, which is implemented using the commercial ABAQUS nonlinear FE software and by providing a listing of the boundary and loading conditions of the IB/FE model and its numerical discretization parameters.

### 2.1. Immersed boundary formulation

In the IB approach to modeling FSI, the momentum, velocity, and incompressibility of the coupled fluid-structure system are described in Eulerian form, whereas the structural deformation, stress, and forces of the immersed solid body are described in Lagrangian form. In this work, the physical domain occupied by the fluid–structure system is denoted 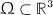, in which **x** = (*x*_1_,*x*_2_,*x*_3_) ∈ Ω are fixed Eulerian (physical) coordinates, and the reference configuration of the immersed solid is denoted 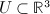, in which **X** = (*X*_1_,*X*_2_,*X*_3_) ∈ *U* are fixed Lagrangian (material) coordinates. ***χ*** : (*U*,*t*) ↦ Ω is a time-dependent deformation mapping relating material and physical coordinates, so that ***χ***(**X**,*t*) ∈ Ω gives the physical position of material point **X** at time *t*. The physical region occupied by the immersed structure at time *t* is ***χ***(*U*,*t*) = Ω_s_(*t*) ⊂ Ω, and the physical region occupied by the fluid at time *t* is Ω_f_(*t*) = Ω ∖ Ω_s_(*t*). The fluid–solid interface in the physical configuration at time *t* is *∂*Ω_s_(*t*) and has an outward unit normal **n**(**x**,*t*) (i.e., **n** is oriented pointing out the solid region Ω_s_(*t*) into the fluid region Ω_f_(*t*)). The boundary of the solid body in the fixed reference coordinate system is *∂U* and has an outward unit normal **N**(**X**).

The continuous equations of motion for the coupled fluid–structure system are 36 as follows: 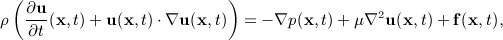
(1)

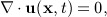
(2)

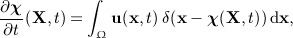
(3)

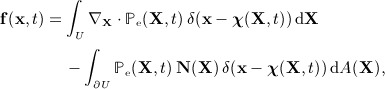
(4)
in which **u**(**x**,*t*) is the Eulerian velocity field that provides a spatial description of the velocity of the coupled fluid–structure system, *p*(**x**,*t*) is the Eulerian pressure field that enforces the incompressibility of the coupled fluid–structure system, **f**(**x**,*t*) is the Eulerian elastic force density, 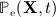 is the first Piola–Kirchhoff elastic stress tensor associated with the immersed solid, and *δ*(**x**) = *δ*(*x*_1_) *δ*(*x*_2_) *δ*(*x*_3_) is the three-dimensional Dirac delta function. Implicit in the formulation (1)–(4) are the assumptions that the mass density *ρ* and viscosity *μ* of the fluid and solid are equal. These assumptions are not essential, however, and versions of the IB method have been developed that permit the use of spatially varying structural mass densities 40–44,39 and viscosities 44,39.

The IB formulation includes two interaction equations that relate Lagrangian and Eulerian quantities via integral transforms with Dirac delta function kernels. The first interaction equation (3) determines the deformations of the immersed solid from the Eulerian velocity field **u**(**x**,*t*). The presence of viscosity ensures that the Eulerian velocity field is continuous, and so Equation (3) implies 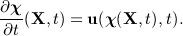
(5) This condition holds along the fluid–solid interface and therefore is equivalent to the velocity continuity (i.e., no slip) condition typically imposed at fluid–solid interfaces by ALE methods for FSI. Because ∇ ⋅ **u**(**x**,*t*) = 0, the immersed solid is automatically treated as an exactly incompressible material. In particular, in the continuous equations, it is not necessary to impose the incompressibility constraint in the Lagrangian equations (e.g., via penalty terms in the elastic energy associated with the immersed solid). However, in practice, we empirically find that such penalty terms improve the stress predictions of the discrete LV model used in this work; see Section 3.

The second interaction equation (4) converts the Lagrangian description of the elastic stresses generated by deformations of the immersed solid into a corresponding Eulerian force density. It can be shown 36 that for **f** defined by Equation (4), the right-hand side of the momentum equation (1) is equivalent to 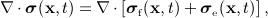
(6)
in which ***σ***(**x**,*t*) is the *total* Cauchy stress tensor of the coupled fluid–structure system, ***σ***_f_(**x**,*t*) is the stress tensor of a viscous incompressible fluid, and ***σ***_e_(**x**,*t*) is the stress tensor that corresponds to the elastic response of the immersed structure. The fluid-like stress tensor ***σ***_f_ is defined by 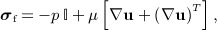
(7)
in which 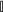 is the identity tensor, and the elastic stress tensor ***σ***_e_ is defined in terms of 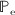 by 
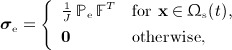
(8)
in which 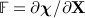 is the deformation gradient tensor associated with the mapping ***χ*** : (*U*,*t*) ↦ Ω and 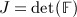 is the Jacobian determinant. The stress tensors ***σ***_f_ and ***σ***_e_ are both defined for all **x** ∈ Ω, and although ***σ***_e_ is nonzero only in the solid region Ω_s_(*t*) ⊂ Ω, fluid-like stresses are present throughout the physical domain. It is possible to remove the viscous stresses within the immersed body via an extension of the present formulation 44,39. Such viscous stresses are appropriate to include in models of many biological tissues, including the ventricular myocardium, in which water comprises a substantial fraction of the tissue volume.

Because ***σ***_e_ is nonzero only within the solid region Ω_s_(*t*), continuity of traction at the fluid–solid interface generally requires discontinuities in the pressure and viscous stress along that interface. Such discontinuities are automatically accounted for in the IB formulation. Let *⟦* ⋅ *⟧* indicate the value of a discontinuity in the bracketed quantity across an interface, which we define for a Eulerian function *g*(**x**) at a position **x** ∈ *∂*Ω_s_(*t*) by 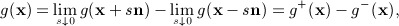
(9)
in which *g*^ + ^(**x**) and *g*^ − ^(**x**) indicate the limiting values of *g*(**x** ′ ) as **x** ′ = **x** ± *s***n** approaches **x** from Ω_f_(*t*) and Ω_s_(*t*), respectively ‡.

Continuity of traction along *∂*Ω_s_(*t*) requires 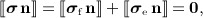
(10)
but the definition of ***σ***_e_ in Equation (8) implies that along the fluid–solid interface *∂*Ω_s_(*t*), 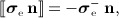
(11)
because ***σ***_e_ ≡ ***0*** in Ω_f_(*t*). Thus, along *∂*Ω_s_(*t*), there must be a corresponding discontinuity in the fluid-like traction, 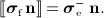
(12)
It can be shown that this implies 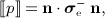
(13)
and 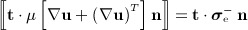
(14)
for any unit vector **t** tangent to the interface. Thus, the limiting value of the elastic traction on *∂*Ω_s_(*t*) determines the magnitudes of the discontinuities in the pressure and viscous stress. By Equation (8) and Nanson's relation, the limiting value of the elastic traction is given by 
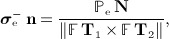
(15)
in which **T**_1_ and **T**_2_ are mutually orthogonal unit tangent vectors to *∂U* in the material coordinate system, and 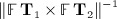 is a conversion factor that relates densities per unit area in the reference and physical configurations at time *t*. 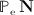 is precisely the force density that appears in the second integral transform in Equation (4), and in the IB formulation, this term imposes the required discontinuities in the pressure and viscous stress 45. Moreover, these jump conditions are equivalent to the traction continuity conditions typically imposed at fluid–solid interfaces by ALE methods for FSI.

### 2.2. Orthotropic constitutive model

We describe the orthotropic elastic properties of the myocardium using a hyperelastic strain-energy functional introduced by Holzapfel and Ogden 10

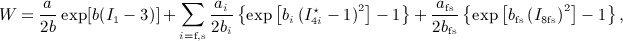
(16)
in which *a*, *b*, *a*_*i*_, and *b*_*i*_ (for *i* = f, s, and fs) are eight non-negative material parameters. Here, 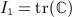 is the first invariant of the right Cauchy–Green deformation tensor 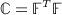, and 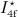, 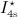, and *I*_8fs_ are structure-based invariants that account for the anisotropy and shear properties of the myocardium. Specifically, let **f**_0_ = **f**_0_(**X**) and **s**_0_ = **s**_0_(**X**) denote the fiber and sheet axes in the reference configuration. In terms of these material direction fields, the basic structure-based invariants are defined by 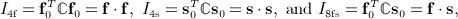
(17)
in which 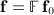 and 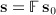 are the deformed fiber and sheet axes in the physical configuration at time *t*. Thus, *I*_4f_ is the square of the stretch in the fiber direction, *I*_4s_ is the square of the stretch in the sheet direction, and *I*_8fs_ measures the relative orientation and deformation in the fiber and sheet directions. The constitutive model is not defined in terms of *I*_4f_ and *I*_4s_ but rather in terms of modified fiber and sheet invariants 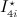, which are given by 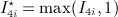
(18)
for *i* = f and s. This ensures that the elastic energies and stresses associated with the collagen fibers are nonzero only in states of extension and not compression. For further discussion on this constitutive model, the reader is referred to the work of Holzapfel and Ogden 10.

The first Piola–Kirchhoff stress tensor is computed from *W* via 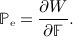
(19)
In this work, we use the constitutive model parameters determined in our previous study 15, *a* = 0.2362* *kPa, *b* = 10.81, *a*_f_ = 20.037* *kPa, *b*_f_ = 14.154, *a*_s_ = 3.7245* *kPa, *b*_s_ = 5.1645, *a*_fs_ = 0.4108* *kPa, and *b*_fs_ = 11.3, which were determined from the porcine experimental data of Dokos *et al*. 46.

### 2.3. Modified stress tensor

As previously discussed, nonzero values of 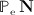 along the fluid–solid interface *∂U* generate discontinuities in the pressure *p* and shear stress *μ*[ ∇ **u** + ( ∇ **u**)^*T*^]. These discontinuities are not artifacts of the IB formulation and are in fact required to obtain traction continuity at the fluid–solid interface. In the spatially discrete IB equations, such pressure discontinuities lead to spurious volume changes, and although these spurious fluxes vanish under grid refinement, they do so only at a first-order rate 47. It is straightforward to verify that with the present constitutive model, 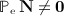 along the fluid–solid interface for 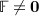.

To reduce the magnitude of the pressure discontinuity in the Eulerian pressure field and thereby reduce the magnitude of spurious volume loss at fluid–solid interfaces, we have found that it is useful to employ a modified stress tensor 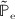 defined so that 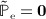 for 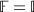. Specifically, we use 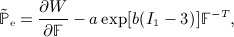
(20)
so that in Ω_s_(*t*), 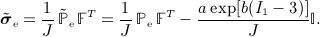
(21)
The modified stress tensor 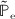 differs from 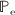 only by a modification to the pressure. Specifically, in the solid region Ω_s_(*t*), the *physical* pressure is 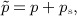
(22)
in which 
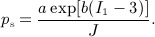
(23)

This modified stress tensor is similar to modifications proposed for simpler neo-Hookean models to ensure that the stress vanishes in the reference configuration 48. We briefly derive this modified stress tensor from a modified strain-energy functional, as follows. Let 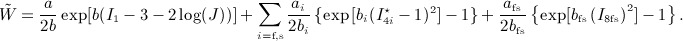
(24)
For an incompressible material, *J* = 1 and log(*J*) = 0, and therefore, 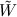 is identical to *W* in Equation (16). In addition, the definitions of *W* and 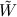 differ only in the isotropic term. Thus, it suffices to consider only the terms 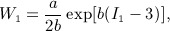
(25)

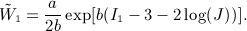
(26)
We compute the corresponding stresses as 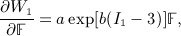
(27)

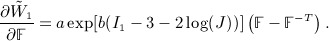
(28)
For *J* = 1, which is exactly imposed in the continuum setting but only approximately imposed in the discrete setting, we have 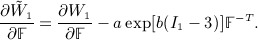
(29)
This is precisely the form of the stress modification employed in this work.

Although incompressibility is enforced in the Eulerian equations (1) and (2), when the continuous equations are discretized, the numerical interpolation of the Eulerian velocity to the solid region may not always yield a divergence-free discrete Lagrangian velocity field. This numerical error vanishes under grid refinement 47, but it can nonetheless lead to non-negligible errors in Lagrangian volume conservation at practical grid spacings, especially in three spatial dimensions. In the present context, such volume conservation errors appear to lead primarily to errors in the computed stress distributions rather than errors in the overall structural displacement. We obtain improved accuracy in the computed stress by including a penalty term to impose approximately the incompressibility constraint in Lagrangian form, namely, 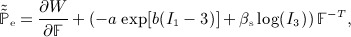
(30)
in which 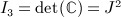 and 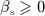 is a penalty parameter. When using this modified stress, the physical pressure in the solid region Ω_s_(*t*) is 
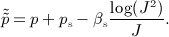
(31)
The Eulerian pressure field *p* is not a state variable of the system and automatically adjusts to account for the inclusion of additional pressure-like terms in the structural stress. Thus, at least in the continuum setting, the dynamics are identical regardless of whether we use 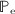 or 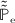 to determine the elastic stress. Except where otherwise noted, we use 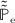 with *β*_s_ = 5.0e6 dyne/cm ^2^ to determine the stress of the body, and we show that in the spatially discrete setting, the modified stress yields improved numerical accuracy (although not order of accuracy).

### 2.4. Numerical methods

The IB formulation (1)–(4) is not well-suited for discretization methods that employ standard FE methods for the structural equations. Consequently, in practice, we use a weak form of the Lagrangian equations: 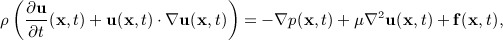
(32)

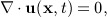
(33)

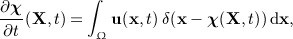
(34)

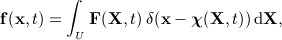
(35)

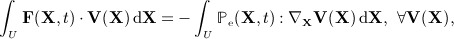
(36)
in which **F**(**X**,*t*) is the Lagrangian elastic force density and **V**(**X**) is an arbitrary Lagrangian test function that is not assumed to vanish on *∂U*. This formulation permits us to approximate the Eulerian equations on a Cartesian grid and to approximate the Lagrangian equations on an unstructured hexahedral FE mesh that corresponds to the imaged LV geometry. We summarize our discretization approach in the following subsections.

#### 2.4.1. Eulerian spatial discretization

Let (*i*,*j*,*k*) label the grid cells of the Cartesian grid used to discretize the Eulerian equations. We denote the physical location of the center of cell (*i*,*j*,*k*) by **x**_*i*,*j*,*k*_ = ((*i* + 1 ∕ 2)*h*,(*j* + 1 ∕ 2)*h*,(*k* + 1 ∕ 2)*h*), in which *h* = Δ*x*_1_ = Δ*x*_2_ = Δ*x*_3_ is the Cartesian grid spacing. We use a staggered-grid discretization of the Eulerian velocity and force fields, so that each vector component is approximated at the center of the Cartesian cell face to which that component is normal. Specifically, the *x*_1_ components of **u** and **f** are approximated at spatial locations 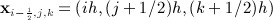, the *x*_2_ components are approximated at spatial locations 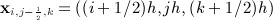, and the *x*_3_ components are approximated at spatial locations 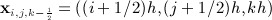. The pressure *p* is approximated at the centers of the grid cells, that is, at spatial locations **x**_*i*,*j*,*k*_. Standard second-order accurate finite difference approximations to the divergence, gradient, and Laplace operators are employed 49,30. The convective term is discretized using a version of the piecewise parabolic method 50,51,49.

#### 2.4.2. Lagrangian spatial discretization

Let *l* label the nodes of the FE mesh used to discretize the Lagrangian equations. The Lagrangian deformation mapping ***χ*** is approximated at the nodes of the FE mesh and is interpolated to arbitrary material coordinates using the FE shape functions, that is, 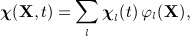
(37)
in which ***χ***_*l*_ is the physical position of FE mesh node *l* and *ϕ*_*l*_ is the nodal basis function associated with node *l*. The deformation gradient is approximated in the element interior by directly differentiating the approximation to ***χ*** in (37), and the stress 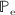 is evaluated directly from the approximation to 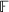. From 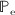, we compute an approximation to the Lagrangian body force **F** that satisfies 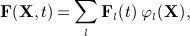
(38)

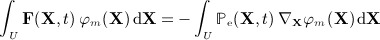
(39)
for all Lagrangian basis functions *ϕ*_*m*_. This implicitly defines a system of linear equations for the nodal force densities **F**_*l*_ involving the FE mass matrix. We use a standard selectively reduced integration approach to avoid volumetric locking 52, whereby reduced-order quadrature rules are used for the pressure normalization (*p*_s_ ≠ 0) and penalty (*β*_s_ ≠ 0) terms in the modified stress, whereas full-order quadrature is used for the remaining terms of the structural stress tensor.

#### 2.4.3. Lagrangian–Eulerian interaction

Our approach to discretize the interaction equations (34) and (35) is first to define a *force-spreading operator* 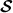 that determines **f** from **F** and then to define the *velocity-restriction operator* 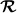 that determines *∂****χ*** ∕ *∂t* from **u** to be the adjoint of the spreading operator, so that 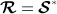. This construction yields a numerical scheme that, at least in the semi-discrete case, conserves energy during Lagrangian–Eulerian interaction 21. In these approximations, we replace the singular delta function kernel with a regularized delta function of the tensor product form *δ*_*h*_(**x**) = *δ*_*h*_(*x*_1_) *δ*_*h*_(*x*_2_) *δ*_*h*_(*x*_2_). In this work, we construct the three-dimensional regularized delta function in terms of the four-point one-dimensional regularized delta function of Peskin 21.

To convert the Lagrangian elastic force density into the corresponding Eulerian elastic force density, we compute **f** = (*f*_1_,*f*_2_,*f*_3_) from **F** = (*F*_1_,*F*_2_,*F*_3_) via 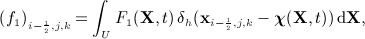
(40)

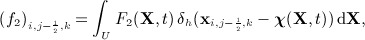
(41)

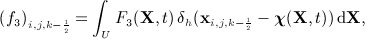
(42)
in which **F** is defined by (38), and the right-hand side integrals are approximated via Gaussian quadrature. We use the notation 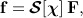
(43)
in which the discrete operator 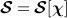 is defined by Equations (40)–(42).

To determine the velocity of the immersed structure, we first note that from (37), **U** = *∂****χ*** ∕ *∂t* satisfies 
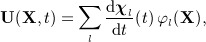
(44)
that is, **U** is in the space of Lagrangian functions spanned by the nodal basis functions and has nodal values **U**_*l*_ = d***χ***_*l*_ ∕ d*t*. It can be shown 38 that the operator 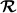 that determines **U** from **u** which satisfies 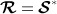 is implicitly defined by requiring **U** to satisfy 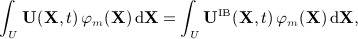
(45)
for all basis functions *ϕ*_*m*_, in which the right-hand side of (45) is computed via the same quadrature rules as used in (38) and in which 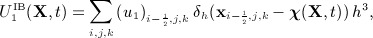
(46)

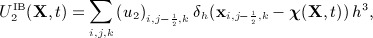
(47)

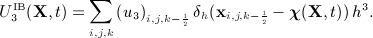
(48)
Notice that **U**^IB^ is a continuous Lagrangian velocity field, but that **U**^IB^ is not in the span of the nodal basis functions. From (44) and (45), it can be seen that **U**(**X**,*t*) is the *L*^2^ projection of **U**^IB^ onto the space of functions spanned by the Lagrangian basis functions. We use the notation 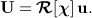
(49)
Applying the operator 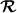 requires the solution of a linear system of equations involving the FE mass matrix, whereas applying the operator 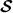 is a purely local operation.

#### 2.4.4. Time stepping

We use a time discretization that combines the explicit midpoint rule for the Lagrangian equations with a Crank Nicolson–Adams Bashforth scheme for the Eulerian equations. Let **u**^*n*^ and ***χ***^*n*^ indicate the approximations to the Eulerian velocity field and Lagrangian deformation field at time *t*^*n*^ = *n*Δ*t*, in which Δ*t* is the time step size, and let 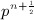 indicate the approximation to the Eulerian pressure field at time 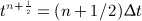. The time discretization proceeds as follows.

First, we compute an approximation to ***χ*** at 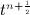 via 
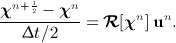
(50)
We compute a corresponding approximation to the Lagrangian force density 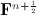 from 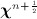, and we spread this force to the Cartesian grid via 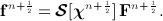
(51)
Next, we solve the incompressible Navier–Stokes equations for **u**^*n* + 1^ and 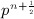 via 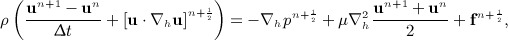
(52)

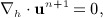
(53)
in which 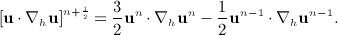
(54)
Solving (52) and (53) requires the solution of the incompressible Stokes equations, and to do so, we employ an iterative Krylov method preconditioned by a block-multigrid preconditioner based on a pressure-free projection method 49. Finally, we determine the structure configuration at time *t*^*n* + 1^ via 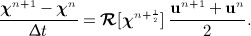
(55)

In the initial time step, we do not have lagged velocity values and therefore cannot compute the multistep approximation to the convective term used in subsequent time steps. Consequently, for the first time step, we instead use an explicit midpoint rule to approximate the convective term, which requires an additional Stokes solve.

#### 2.4.5. Implementation

This IB/FE scheme is implemented within the open-source IBAMR software framework 53,27,28, which provides an adaptive and distributed-memory parallel infrastructure for developing FSI models that use IB method. IBAMR leverages functionality provided by other freely available software libraries, including SAMRAI 54,55, PETSc 56–58, and libMesh 59,60.

### 2.5. Imaging-derived human left ventricle model

**Figure 1:**
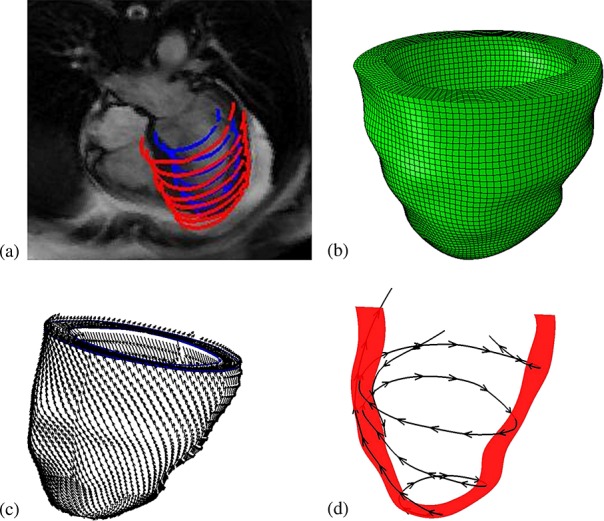
Imaging-derived model of the human left ventricle (LV). (a) Manual LV boundary segmentation; (b) hexahedral mesh; (c) rule-based fiber architecture; and (d) fiber tracing.

A human left ventricular model is reconstructed from a cardiac MR imaging study on a healthy volunteer (male, age 28 years), performed at the British Heart Foundation MRI Facility at the University of Glasgow, as described previously 15; see Figure 1(a). In this work, we use a hexahedral LV mesh composed of 48,050 elements and 53,545 nodes; see Figure 1(b). A rule-based myocardial fiber generation procedure based on the work of Potse *et al*. 61 is employed to determine the fiber and sheet directions within the myocardium. In the present work, the fiber angle rotates from − 60° to 60° from endocardium to epicardium, and the sheet angle rotates from − 45° to 45°, thereby corresponding to a normal, healthy LV 11; see Figure 1(c,d).

### 2.6. Structural analysis by ABAQUS

To verify the implementation of the IB/FE model, the same structural model is also analyzed using commercial ABAQUS (SIMULIA, Providence, RI, USA) nonlinear FE software, as in earlier work 15,16.

### 2.7. Physical and numerical parameters, boundary conditions, and loading conditions

In our simulations, we set *ρ* = 1.0 g/ml and *μ* = 0.04 cP §, and Ω is taken to be a 15 cm × 15 cm × 15 cm box that is discretized on a regular Cartesian grid. The integrals of the interaction equations are discretized using dynamically generated Gaussian quadrature rules that ensure a density of at least two quadrature points per Cartesian mesh width. A Cartesian grid convergence analysis is performed to demonstrate the sensitivity of the results on the Eulerian spatial mesh width. We consider *N* × *N* × *N* Cartesian grids for *N* = 64, 80, 96, 112, and 128. Unless otherwise noted, in the majority of our simulations, we use *N* = 96, which we find to yield essentially grid-converged results; see Section 3. In this case, the Eulerian grid spacing is *h* = Δ*x*_1_ = Δ*x*_2_ = Δ*x*_3_ = 0.15625* *cm. A relatively small time step size is required because of the explicit time stepping scheme employed herein. Using the method of bisection, we empirically determined that a time step size of Δ*t* = 1.6276e-4 s is approximately the largest stable time step size permitted by our method and model.

**Figure 2:**
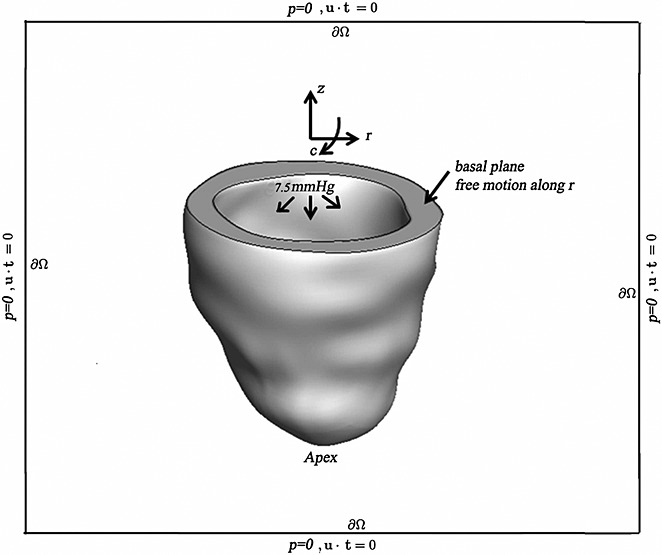
Schematic illustrating the boundary and loading conditions applied to the IB/FE LV model. *c*, circumferential direction; *r*, radial direction; and *z*, axial direction. LV cavity pressure loading is applied to the endocardial surface, and displacements of the basal plane are fixed in the *c* and *z* directions, thereby permitting only radial expansion. The overall computational domain is represented by the black box with zero pressure and zero tangential slip along *∂*Ω, in which u is the Eulerian velocity field and t is the unit tangent vector along *∂*Ω.

In both the IB/FE-based model and the FE-based model, longitudinal and circumferential displacements of the basal plane are constrained, whereas radial displacements of the basal plane are left free ¶ . The reminder of the left ventricular wall, including the apex, is also left free. A spatially uniform pressure load is applied to the endocardial surface. In the IB/FE model, the endocardial pressure linearly increases over a period of 1.0 s from 0 to 7.5 mmHg and is subsequently maintained at 7.5 mmHg until the model reaches steady state. The static FE model determines the deformations only for the final loading pressure. In the IB/FE model, displacement-type boundary conditions are approximately imposed via penalty forces that provide an energetic penalty for the constrained motions, whereas in the ABAQUS-based FE model, the displacement boundary conditions are imposed exactly. Additionally, in the IB/FE model, zero tangential slip and zero normal traction boundary conditions are imposed along *∂*Ω. For an incompressible flow, imposing no-slip tangential velocity boundary conditions along a flat boundary implies that the normal component of the boundary traction (i.e., **n** ⋅ ***σ***_f_ ⋅ **n**) is proportional to the pressure 62. Thus, the physical boundary conditions imposed on the exterior boundary of the computational domain are equivalent to a boundary condition for the pressure. Unless otherwise noted, these boundary and loading conditions are employed in all computations described herein. Figure 2 shows a schematic of the boundary and loading conditions for the LV model. To compare the IB/FE model predictions to results from the ABAQUS-based FE model, we allow the dynamic IB/FE model to run until it reaches equilibrium, which occurs at approximately 5 s.

## 3. RESULTS

We first perform a grid sensitivity analysis for *N* × *N* × *N* Cartesian grids for *N* = 64, 80, 96, 112, and 128. Table1 shows the differences in the computed displacements, fiber strains, and fiber stresses when compared with the ABAQUS-based model. Notice that only small differences in the computed values are observed for 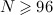. Consequently, we use *N* = 96 for all subsequent IB/FE computations.

**Table 1:** Cartesian grid sensitivity analysis, comparing the mean displacement, fiber strain, and fiber stress differences related to the results from the ABAQUS-based model. Notice that essentially grid-resolved results are obtained for 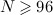.

*N*	Displacement (mm)	ln(*λ*_f_) (Dimensionless)	*σ*_ff_ (kPa)
64	0.59 ± 0.47	(0.9 ± 6)e-4	− 0.7 ± 4
80	0.13 ± 0.07	(0.1 ± 5)e-5	− 0.2 ± 2
96	0.07 ± 0.02	(0.27 ± 4)e-4	− 0.12 ± 0.7
112	0.07 ± 0.02	(0.24 ± 6)e-4	− 0.13 ± 0.7
128	0.07 ± 0.02	(0.25 ± 6)e-4	− 0.12 ± 0.6

ln(*λ*_f_), logarithmic fiber strain; ***σ***_ff_, fiber stress.

**Figure 3:**
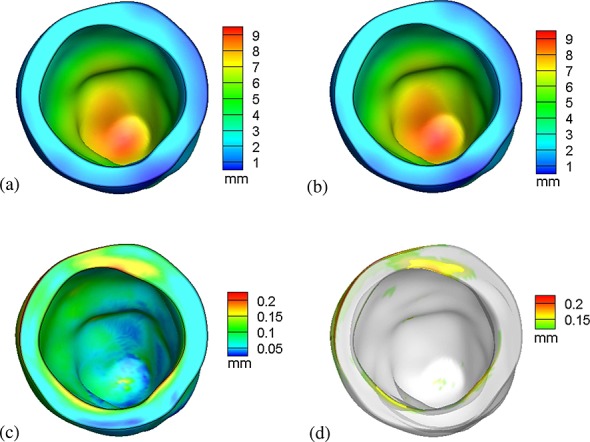
Distributions of displacement for (a) the IB/FE model and (b) the ABAQUS-based FE model; (c) norm of the difference in displacements produced by the two models; and (d) regions in which the normed difference is greater than the mean difference plus two standard deviations.

The displacement magnitudes determined by both models at a loading pressure of 7.5 mmHg are shown in Figure 3 panels (a) and (b), whereas panels (c) and (d) show the normed difference in the computed displacement. The maximum displacement obtained using the IB/FE model is 9.31 mm, which is within 1% of the maximum displacement of 9.40 mm obtained from the ABAQUS-based FE model. In addition, the spatial distributions of displacement are nearly identical in both models; compare panels (a) and (b) of Figure 3. Figure 3(c) plots the spatial distribution of the norm of the difference of the displacement vectors. In most of the volume, the differences are seen to be negligible, much less than 0.1 mm, that is, much less than a 1% difference. Figure 3(d) shows the spatial distribution of the regions where the displacement difference is greater than the mean difference plus two standard deviations. From Figure 3(d), it is clear that the regions with the greatest differences are mainly located along the basal plane, where the displacement boundary conditions are applied.

**Figure 4:**
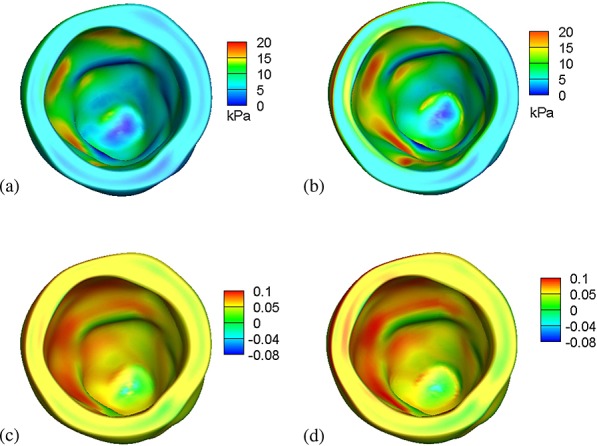
Fiber stresses generated by (a) the IB/FE model and (b) the ABAQUS-based FE model, and logarithmic fiber strains generated by (c) the IB/FE model and (d) the ABAQUS-based FE model.

The distributions of the fiber stress ***σ***_ff_ are shown in Figure 4(a,b) for both the IB/FE and FE models. Figure 4(c,d) show the logarithmic fiber strain distributions for both the IB/FE and FE models. Stress and strain distributions generated by the two models are similar, although some minor differences can be seen. For instance, along the basal plane, there are modest differences, which likely result from differences in the treatment of displacement-type boundary conditions in the two models.

**Figure 5:**
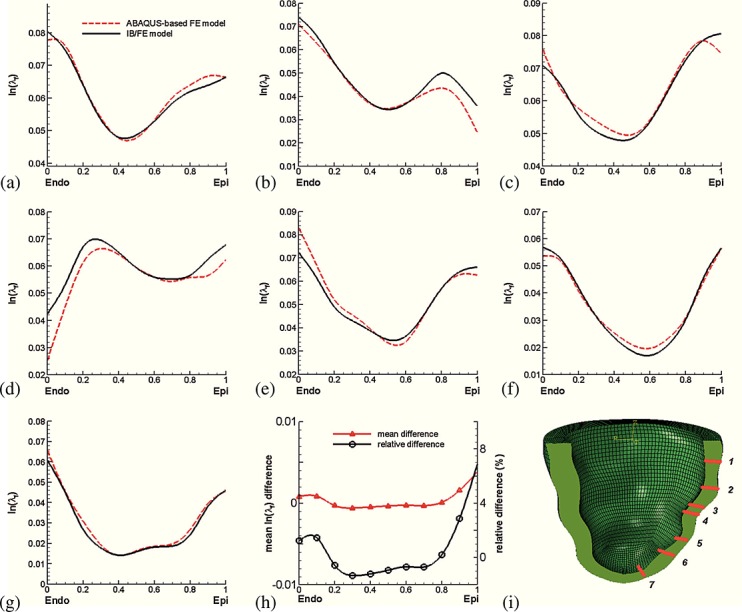
(a–g) Transmural distributions of logarithmic strain ln(*λ*_f_) along seven selected transmural paths; (h) transmural average strain difference between the IB/FE and ABAQUS-based FE models, in which the average difference from the seven paths is in red and the relative difference is in black; and (i) definitions of the seven selected material paths.

**Figure 6:**
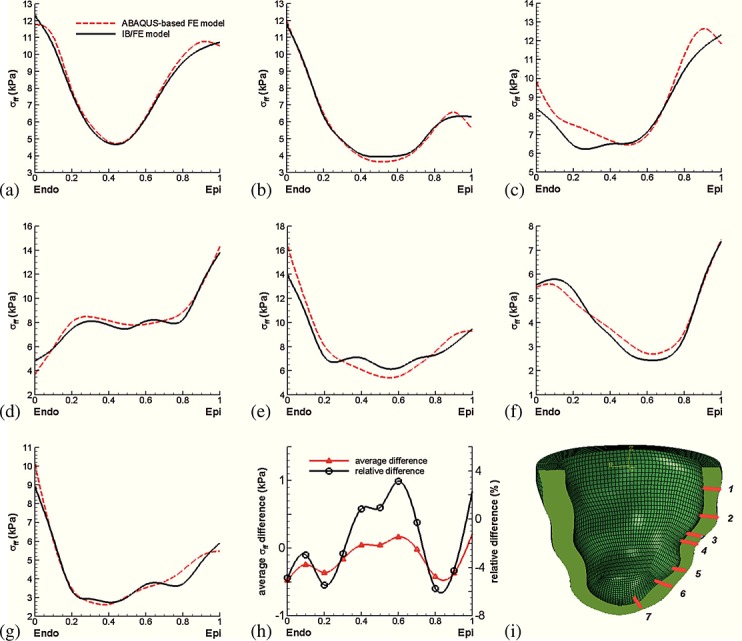
(a–g) Transmural distributions of fiber stress *σ*_ff_; (h) transmural average stress difference between the IB/FE and ABAQUS-based FE models, in which the average difference is in red and the relative difference is in black; and (i) definitions of the seven selected material paths.

To facilitate a closer inspection of the results, seven selected transmural paths across the LV free wall are constructed, as shown in Figure 5. Figure 5(a–g) show fiber strain comparisons along the seven paths, and Figure 5(h) shows the differences between the IB/FE and FE-based models along those selected paths. The results indicate that the general fiber strain distributions obtained from both models are similar; however, the IB/FE model tends to yield slightly larger fiber strains close to the epicardium. The relative strain difference is generally less than 8%; see Figure 5(h). Figure 6(a–g) show fiber stress distributions along the same seven paths. Again, similar results are found in Figure 5: the fiber stress is similar in the two models, and the relative fiber stress difference is less than 8%.

**Figure 7:**
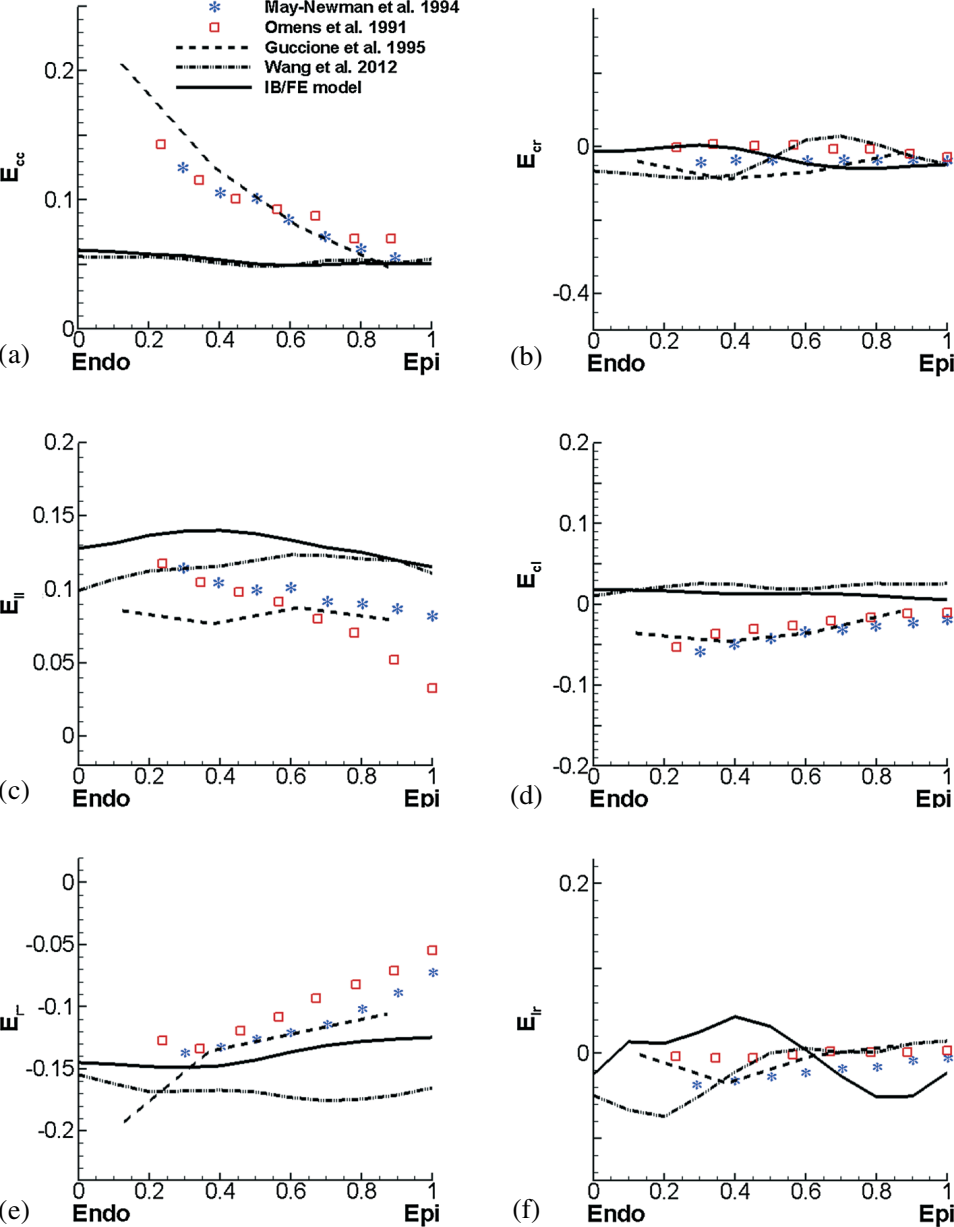
Transmural distributions of normal and shear strains along the equatorial region of the anterior wall. Results from the IB/FE-based and FE-based models are compared with experimental studies of Omens *et al.* 64 and May-Newman *et al.* 63 and numerical results from a canine left ventricular model of Guccione *et al.* 12. *E*_cc_, circumferential strain; *E*_rr_, radial strain; *E*_ll_, longitudinal strain; *E*_cr_, circumferential–radial shear; *E*_cl_, circumferential–longitudinal shear; *E*_lr_, longitudinal–radial shear.

Figure 7 compares the results of the IB/FE-based and FE-based models to published strain distributions from earlier experimental and computational studies of the canine heart 63,12,64. These data show that there is an overall qualitative and quantitative agreement between the IB/FE model predictions, the FE-based model, and the earlier experimental and computational results, especially for *E*_rr_ (radial strain), *E*_cr_ (circumferential–radial shear), and *E*_lr_ (longitudinal–radial shear). The IB/FE-based and FE-based models are also in good quantitative agreement for *E*_cc_ (circumferential strain), *E*_ll_ (longitudinal strain), and *E*_cl_ (circumferential–longitudinal shear), although in those cases, there are some discrepancies between our models and the results from earlier experimental and computational models. We remark that the strain distributions determined by the FE model in this work are moderately different from those determined in our earlier work 15, in which different boundary conditions were imposed on the basal plane of the model LV. This suggests that these strain measurements are relatively sensitive to changes in boundary conditions.

**Figure 8:**
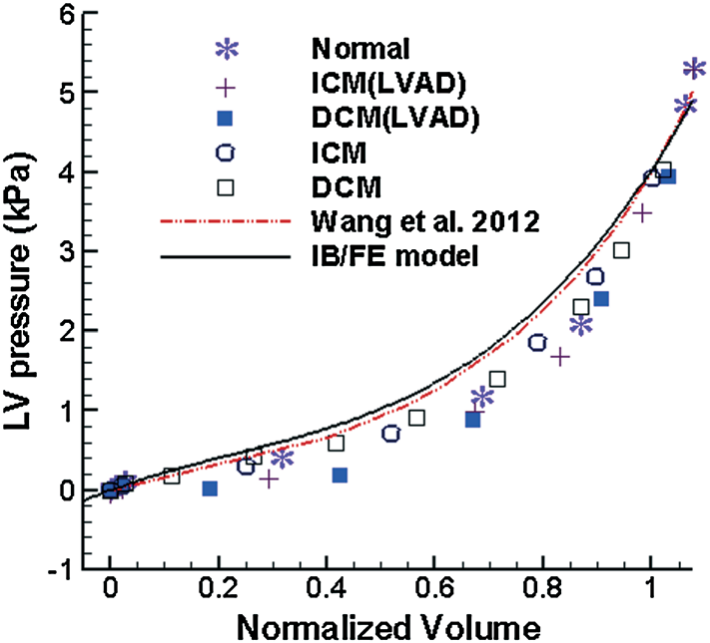
Comparison of end-diastolic pressure–volume relationships obtained using the IB/FE model and the FE results of Wang *et al.* 15 to measurements from *ex vivo* human hearts 65. Normal, healthy heart; ICM, ischemic cardiomyopathy; DCM, diopathic dilated cardiomyopathy; LVAD, hearts supported by a left ventricular assist device.

Figure 8 compares the end-diastolic pressure–volume relationship (EDPVR) of the IB/FE and FE models to EDPVR measurements from *ex vivo* human hearts by Klozt *et al.* 65. These curves are normalized by EDV_*n*_ = (EDV − *V*_0_) ∕ (*V*_30_ − *V*_0_), in which *V*_0_ is the initial unloaded LV volume, and *V*_30_ is the LV volume with a loading of 30 mmHg. It is clear that there is good agreement between the IB/FE and FE model results and the data of Klozt *et al*.

**Figure 9:**
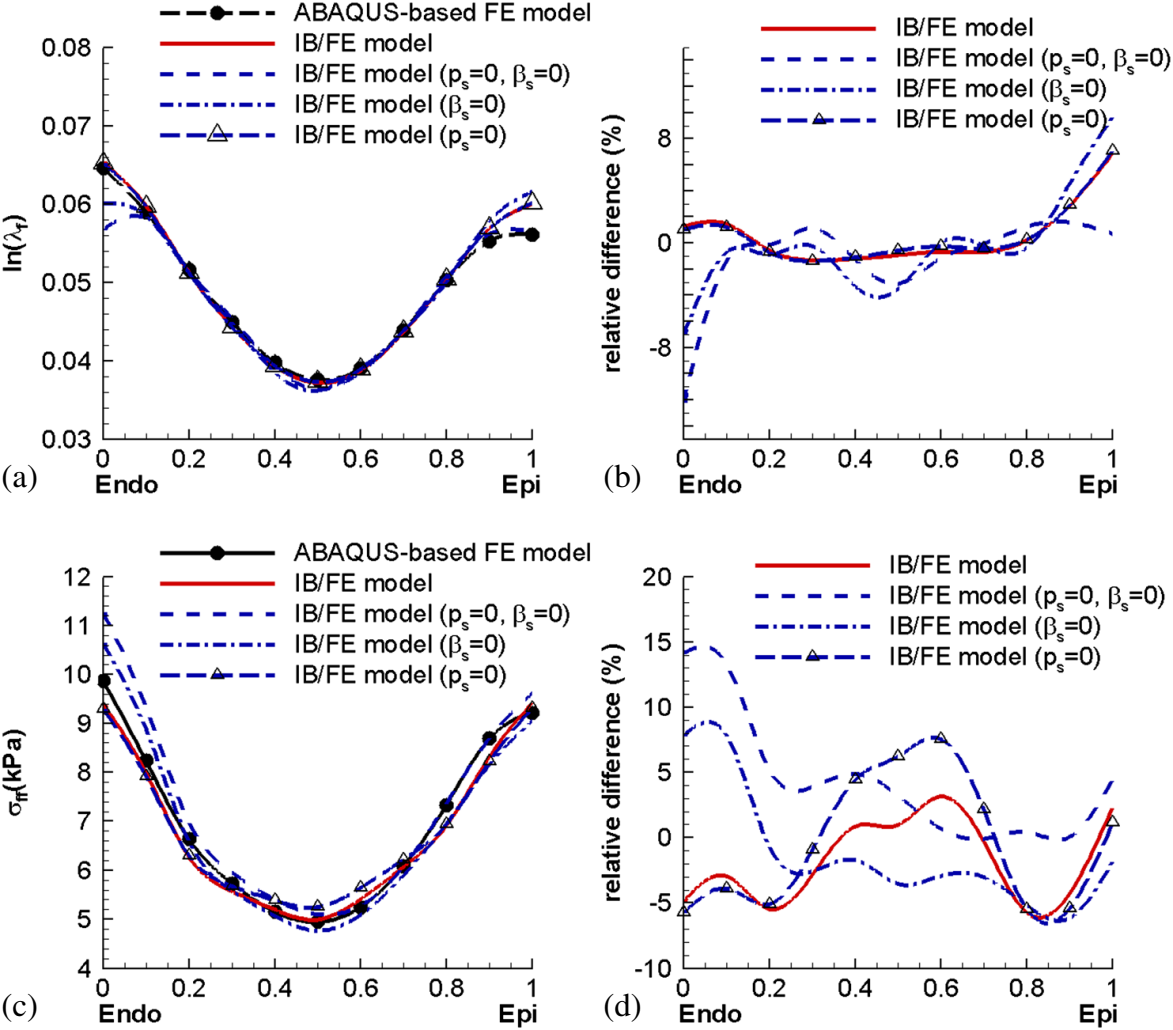
Comparisons of the average logarithmic fiber strain and fiber stress between the ABAQUS-based FE and IB/FE models using the modified structural stress tensor 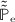, with or without the pressure shift *p*_s_ and incompressibility penalty term. The average stress or strain is defined as the average value over all of the selected paths.

Figure 9 shows the effects of using the modified structural stress tensors, which include an additional pressure-like term *p*_s_, an incompressibility penalty term that is activated for *β*_s_ > 0, or both, on the average logarithmic fiber strain and the stress along the selected seven paths, as defined in Figure 5(i) and their differences. For the fiber strain, the differences are mainly concentrated at the endocardial and epicardial surfaces, where the IB/FE method yields lower-order accuracy because of its treatment of the Eulerian pressure discontinuities at those surfaces. A modified structural stress tensor obtained when using only the penalty term (i.e., *p*_s_ = 0 and *β*_s_ ≠ 0) predicts similar fiber strains as when we include both pressure normalization and the penalty term (i.e., *p*_s_ ≠ 0 and *β*_s_ ≠ 0); see Figure 9(b). By contrast, higher accuracy is achieved in the fiber stress when including both the pressure normalization and the penalty term, as shown in Figure 9(d). When these terms are not included in the stress, the IB/FE method yields fiber strains that are as much as 12% lower than those generated by the ABAQUS-based model and fiber stresses that are as much as 14% greater than those generated by the ABAQUS-based model. Table 2 summarizes the effects of different modifications to structural stress tensors on the computed incompressibility of the myocardium. By including *β*_s_ ≠ 0 in the structural stress tensor, *J* is very close to 1, indicating near incompressibility, whereas with *β*_s_ = 0, some elements can be compressed substantially. Table 2 suggests that for the discretized LV model used in this study, it is necessary to include terms that act to impose incompressibility in a Lagrangian sense even though the computed Eulerian velocity field is discretely divergence free.

**Table 2:** The effects of including pressure normalization (*p*_s_ ≠ 0) and volumetric penalty terms (*β*_s_ ≠ 0) in the structural stress tensor on myocardial incompressibility at the integration points of the reduced-order quadrature rule.

Pressure normalization	Volumetric penalty	Min *J*	Max *J*
Yes	Yes	0.9937	1.008
Yes	No	0.1540	1.141
No	Yes	0.9932	1.008
No	No	0.2728	1.093

Figure 10 shows the velocity fields at *t* = 0.75 s and *t* = 5.0 s. At the beginning of the simulation, when the ventricular wall is initially pressurized, there is inflow into the LV as the chamber expands. By time *t* = 5.0 s, the model has essentially reached equilibrium and there is no further flow. Because of the lack of valves and simplified boundary conditions, the flow patterns from the present model are not physiological.

**Figure:**
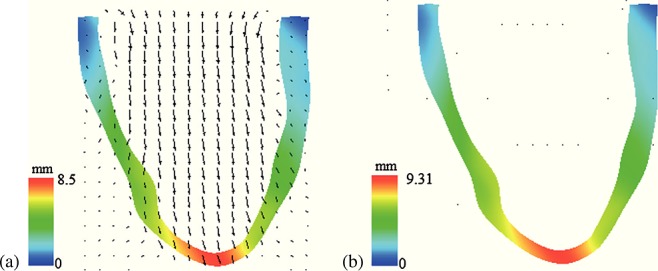
Velocity field (scaled by magnitude) and structural configuration (colored by displacement magnitude) along a plane bisecting the chamber (a) at *t* = 0.75 s and (b) at *t* = 5.0 s. The maximum velocity is 41 mm/s in panel (a) and 3 mm/s in panel (b).

## 4. DISCUSSION

The IB method has been widely used to model cardiac FSI and to predict flow patterns and structural deformations; however, the fiber-based structural models conventionally used in IB simulations of cardiac dynamics are restricted to somewhat simple constitutive models. This makes it difficult to incorporate experimentally based material models into IB computations and thereby limits the ability of these models to predict tissue stress and strain distributions. Quantities such as the end-diastolic left-ventricular stress and strain distributions in patients with diastolic heart failure are important biomarkers and, moreover, are not accessible via standard clinical imaging. A typical approach to obtain these stress and strain data is to employ a nonlinear FE method along with a detailed model of the myocardium 15,16,14,10. Herein, we have demonstrated that the IB/FE framework provides an effective alternative approach to conventional FE methods that yields strain and stress distributions that are comparable with those generated by conventional FE methods for large deformation structural mechanics.

The primary focus of this study is to verify and to validate the stress and strain predictions generated by the IB/FE framework by comparisons with results obtained using a previously described LV model implemented within the ABAQUS FE software and by comparisons with earlier computational and experimental data sets. For the purposes of these comparisons, it is necessary to use quasi-static conditions as well as loading conditions and, where possible, material properties corresponding to those used in these earlier studies. Our results are some of the first to consider the accuracy of the stress and strain distributions generated by the IB method for realistic models of ventricular mechanics, and they indicate that the IB method can yield quantitatively accurate stresses and strains for such models. We show that improvements in the accuracy of the stress predictions can be obtained by a simple modification to the Lagrangian stress tensor that acts to reduce the magnitude of discontinuities in the Eulerian pressure field. We further show that obtaining high accuracy can require imposing the incompressibility constraint in both Lagrangian and Eulerian forms. This latter result is contrary to the prevailing approach used within the IB community, whereby it is typically assumed that it is sufficient to impose incompressibility only as an Eulerian constraint and not also as a Lagrangian constraint 21.

Although good agreement is demonstrated between the IB/FE-based model and the benchmark FE model implemented in the ABAQUS FE software, the strain and stress results generated by the current model via either approach need to be interpreted with some care because of limitations of the model: (i) the material parameters are not subject specific but rather are obtained from a porcine study 46; (ii) the endocardial pressure loads are uniform and are no subject-specific; (iii) the heart geometry is simplified and does not include a right ventricle, the atria, the valves, and the great vessels; (iv) LV biomechanics under diastolic loading is modeled as purely passive inflation, as is commonly done in the literature 13; and (v) experimental results from different animal models are used for strain comparison as in Figure 7.

We remark that the present model also does not account for the dynamics of the diastolic phase of the cardiac cycle. Instead, it yields stress and strain distributions that correspond to late diastole (after diastasis 66), when the heart has been passively filled and the heart is approximately in an equilibrium state, or to *ex vivo* conditions in which the heart is externally pressurized, as in the experiments described in 64,63,65. A more complete model of the diastolic phase of the cardiac cycle would include the rapid filling, slow filling, and atrial contraction phases of diastole. Such a model would require a description of cardiac dynamics. Moreover, obtaining the correct stress and strain states within the LV myocardium at the onset of diastole would seem to require a model of myocardial active tension generation and relaxation, as well as a description of both the strain state and the activation state at the onset of diastole. Although these features can all be incorporated into the present modeling framework, they are beyond the scope of this study, which focuses on verifying and validating the computational predictions of this IB/FE model under typical diastolic pressure loads.

In addition, there remain some relatively small discrepancies between the IB/FE-based and FE-based models. For example, the maximum displacement obtained from the IB/FE model is within 1% of that obtained using the FE-based model. Fiber stresses and strains along the selected transmural paths across the left ventricular free wall are also slightly different. Stress and strain are derived quantities in both the IB/FE-based and FE-based models, and it is well known that differences in displacements can lead to differences in the recovered stress or strain that are an order of magnitude larger 67. Small differences in the computed displacements will result from different numerical treatments of the equations of structural mechanics by the IB/FE implementation and by ABAQUS and from differences in the manner in which displacement boundary conditions and pressure loading conditions are imposed in the two models. In addition, different stress recovery methods are used in the two codes. Because detailed experimental strain and stress measurements inside the LV wall are currently lacking, it is not possible to assess the actual prediction accuracy of either simulation approach, only to verify that both approaches yield comparable model predictions.

Although we only consider quasi-static conditions in this work, the IB method is well-suited for fully dynamic FSI models of the heart wall and the blood. Indeed, the IB method is primarily intended to simulate dynamics. In particular, because our IB/FE model must use a time step that is below the stability threshold associated with the explicit time stepping scheme used in our present implementation, the IB/FE model requires substantially more computational time than the comparable FE model. The IB/FE method is designed to model FSI, however, and we expect that it will be relatively straightforward to extend this model to yield subject-specific simulations of cardiac dynamics with potential clinical applications. We aim to consider the fully dynamic case in future work and to incorporate more realistic loading conditions and material models with active contraction for human ventricular tissue.

## 5. CONCLUSIONS

We have successfully applied an IB method with FE mechanics to an imaging-derived subject-specific model of the human LV under diastolic loading conditions. The predicted stress and strain distributions agree well with our previous model, which used a conventional nonlinear FE method, as well as other experimental and computational data sets that serve to validate both the IB/FE-based and FE-based models. These results indicate that it is possible to obtain quantitatively accurate results using the IB method in conjunction with realistic experimentally constrained constitutive models and suggest that the IB method can also yield quantitative accuracy when using such constitutive models in the context of fully dynamic models of the heart.

## Footnotes

‡ Recall that **n** is oriented so that it points from the solid region Ω_s_(*t*) into the fluid region Ω_f_(*t*).

§ In this study, we consider only the equilibrium configuration of the LV model under steady loading conditions, and although the values of *ρ* and μ affect the transient dynamics, they have no effect on the steady-state results.

¶ These boundary conditions are slightly different than those used in our earlier work 15,16. In those earlier studies, longitudinal displacements of the basal plane were constrained along with circumferential displacements of the epicardial edge of the basal plane. Except where otherwise noted, all FE-based model results described in this work use the new boundary conditions, that is, longitudinal and circumferential but not radial, displacements are constrained along the basal plane.
